# Persistent Tn polyagglutination syndrome during febrile neutropenia: a case report and review of the literature

**DOI:** 10.1186/1752-1947-5-8

**Published:** 2011-01-14

**Authors:** Arturo Loaiza-Bonilla, Daniel Horowitz, Sheenu Sheela, Anupa Baral, Gabriel Tinoco, Christos Kyriakopoulos

**Affiliations:** 1 The Sidney Kimmel Comprehensive Cancer Center, Johns Hopkins University, Baltimore, Maryland 21287, USA

## Abstract

**Introduction:**

Tn polyagglutination syndrome is a rare disorder that has been reported on only a few occasions in the literature, and, to the best of our knowledge, never before in the context of febrile neutropenia.

**Case presentation:**

We report the case of a 26-year-old Caucasian woman who presented to our emergency department complaining of a persistent fever over the previous three days. She had a history of long-standing refractory pancytopenia with multi-lineage dysplasia and severe neutropenia, but she had rarely experienced infection. The results of a physical examination and multiple laboratory tests were unremarkable. While investigating the possible causes of the refractory, long-standing pancytopenia, the possibility of a polyagglutinable state was suggested. Blood samples were sent to the laboratory for an analysis of mixed-field seed lectin agglutination assay. A serum lectin panel confirmed the final diagnosis of Tn-activation.

**Conclusions:**

We should include Tn-activation in our differential whenever we encounter cases of refractory long-standing idiopathic cytopenias and inconclusive bone marrow results displaying multi-lineage dysplasia. Novel genetic techniques have recently revealed the interesting pathophysiology of this phenomenon. The recognition and inclusion of Tn polyagglutination syndrome in our differential diagnoses has important clinical implications, given its main associated features, such as severe thrombocytopenia and neutropenia, which are usually linked to a benign clinical course and prognosis. Increased awareness of the polyagglutinable disorders will potentially decrease the need for invasive and costly medical interventions and also raises the need for monitoring of this specific sub-set of patients. In addition, the study of the expression and implications of Tn, and other similar antigens, offers a fascinating perspective for the study of its role in the diagnosis, prognosis and immunotherapy of solid tumors and hematological malignancies. The infrequency with which Tn polyagglutination syndrome is encountered, its clinical features and its pathophysiology make it a formidable diagnostic challenge.

## Introduction

During an initial assessment of patients with unexplained neutropenia, clinicians should include Tn polyagglutination syndrome (TnP) in their differential diagnoses. Many cases remain undiagnosed due to a lack of knowledge about this entity, its implications and its pathophysiology. Our case report is intended to increase awareness of this diagnosis. We report the case of a patient with persistent TnP and undertake a concise review of the literature regarding this condition.

## Case presentation

A 26-year-old Caucasian woman presented to our emergency department complaining of a persistent fever (ranging between 38.5°C and 40°C) over the previous three days. She had been in her usual state of health until this time. She also complained of palpitations, chills and diaphoresis. She denied any other symptoms. She described no known contact with sick individuals, no trauma, insect bites or any history of travel.

**Figure 1:**
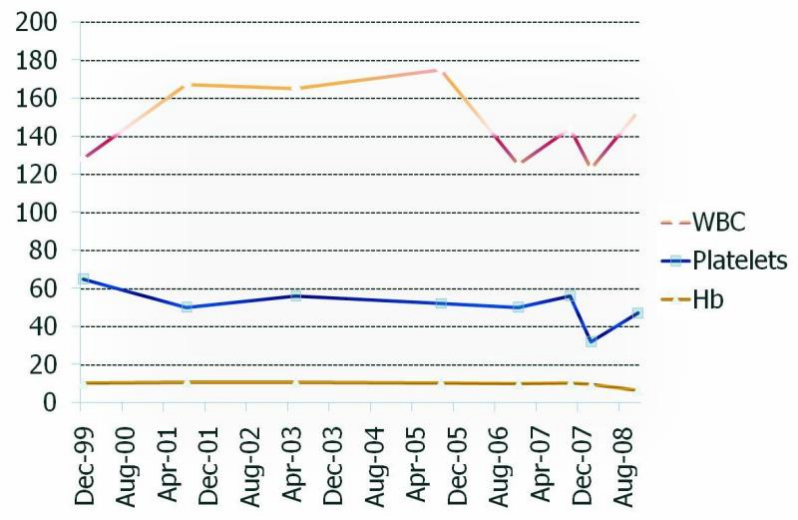
**Complete blood count levels**. Units: WBC (×10^1 ^cells per mm^3^); Hb (g/dL); Platelets (×10^3 ^cells per mm^3^)

She was born full term to a 24-year-old mother via vaginal delivery, weighing 7 lbs 3oz. At three-weeks of age, she developed diffuse petechiae which persisted and, after multiple studies, she was diagnosed as having a long-standing refractory pancytopenia with multi-lineage dysplasia. Her average blood count values were a white blood cell (WBC) count of 1.5×10^3 ^cells per mm^3 ^with severe neutropenia, with an approximate absolute neutrophil count (ANC) in the 400 range. Her hemoglobin level was 8 g/dL and her platelet count was approximately 45×10^3^/mm^3^. Despite her condition, she had an infrequent history of infections. These included otitis media and adenitis at eight months of age, *Staphylococcal cellulitis *at 15 months and two instances of uncomplicated pyelonephritis at ages 15 and 21 which were treated successfully with amoxicillin and gatifloxacin. She had two uneventful pregnancies and a third which was complicated by severe pre-eclampsia. She noted occasional petechiae and some bruising, but had not had any serious hemorrhages. Please refer to Figure 1 for a graphic description outlining the 10-year span blood count numbers for this patient.

She has had a bone marrow biopsy performed every two years as a follow-up procedure for her refractory pancytopenia, and no clear etiology has been defined. This lack of diagnostic certainty and her ongoing concerns regarding her future health and that of her children has caused her major distress. She has also been told by some physicians that she and her family may carry an increased risk for hematological malignancies.

On physical examination, she was febrile with a temperature of 38.3°C. Her heart rate was 105 beats per minute, her blood pressure was 115/82 mmHg and her respiratory rate was 17 breaths per minute with an oxygen saturation of 99 percent at room air. Her physical examination was unremarkable except for sinus tachycardia. On her admission to our hospital, our laboratory studies showed a WBC count of 1.53×10^3 ^cells per mm^3 ^with 20 percent neutrophils and 66 percent lymphocytes. Her ANC was 400, her hemoglobin level was 6.8 g/dL, her Mean Corpuscular Volume was 82 fL and her platelet count was 47×10^3^/mm^3^. Her electrolytes were within normal range. Her prothrombin time was 19.1 seconds, and the International Normalized Ratio -INR- was 1.1.

She was diagnosed with febrile neutropenia and she was started on empiric IV piperacillin/tazobactam after we obtained blood and urine cultures. A computed tomography (CT) scan of her chest, paranasal sinuses, abdomen and pelvis were ordered to evaluate the source of the fever. All the imaging studies showed no abnormalities.

On day two, her blood count values still remained low. After the ingestion of an oral contrast, she developed soft stools which prompted us to undertake stool studies. These were negative for lactoferrin, *Clostridium difficile *and fecal leukocytes. Assessment for ova and parasites as well as a stool culture were also negative. A direct Coombs test was negative, her reticulocyte count was 0.2 percent, and an iron study panel revealed serum iron levels of 7 mmol/L, ferritin 72 ug/L, and iron saturation levels of four percent. Her lactate dehydrogenase and bilirubin levels were unremarkable.

She continued to spike fevers overnight with no evident source of infection. A bone marrow biopsy was completed, suggesting once again the diagnosis of refractory pancytopenia with multi-lineage dysplasia. No increased blasts were noted. Fluorescent *in situ *hybridization (FISH), flow cytometry and cytogenetics did not reveal any major abnormalities. While investigating possible causes of the refractory, long-standing pancytopenia, the possibility of a polyagglutinable state was suggested. Blood samples were sent to the laboratory for an analysis of mixed-field seed lectin agglutination assay. A serum lectin panel confirmed the final diagnosis of Tn-activation (*Dolichos biflorus +, Glycine soja +, Salvia sclerea *+) and TnP was diagnosed. She responded to treatment very well and after 48 hours of being afebrile, she was discharged with a five day course of cefpodoxime. Her samples were sent for Tn monoclonal antibody immunochemistry which reconfirmed her diagnosis.

TnP is a rare disorder that has been reported on only six occasions in the medical literature and never before in the setting of febrile neutropenia. TnP is an acquired disorder, characterized by the defective biosynthesis of red blood cell (RBC) membrane glycoproteins that results in the exposure of normally cryptic N-acetylgalctosamine residues (GalNAc) [1,2].

**Figure 2:**
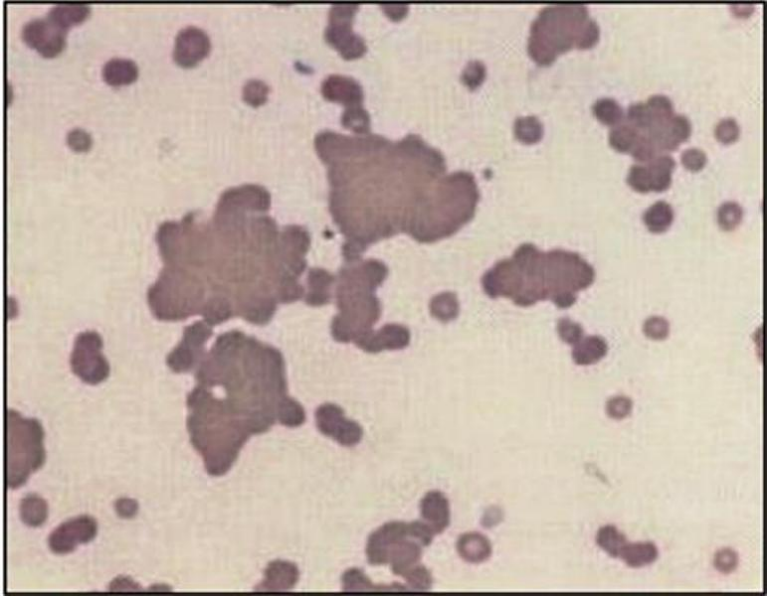
**RBC polyagglutination**. Peripheral smear displaying polyagglutination phenomenon

Polyagglutination is the term applied to RBC that are agglutinated by almost all samples of human sera from adults, but not by autologous serum or sera of newborns [1-3] (Figure 2). Previously, all cases of TnP, and many other polyagglutinable phenomenons, were diagnosed during infancy or at the moment when a blood group classification for a transfusion was made, as former techniques of hemoclassification were performed using human adult serum containing multiple antibodies. However, current blood grouping practice uses murine diluted monoclonal antibody re-agents that do not include any other potentially pro-agglutinating immunoglobulins (Ig), thus denying the opportunity to recognize polyagglutinable cells and their implications [4,5]. This has been reflected in a lack of reports about this condition over the last 30 years.

Tn RBC are polyagglutinable because most adult sera contain naturally occurring anti-Tn. T and Tn antigens (from Hiibener-Thomsen-Friedenreich) are normally inaccessible to the immune system. These antibodies occur primarily due to the exposure to the intestinal flora, where highly immunogenic T and Tn-specific structures are present in most *Enterobacteriaceae *[1-4]. The Tn defect can also be found on the membranes of platelets, granulocytes and lymphocytes [6]. The first example of TnP was encountered in a patient with hemolytic anemia, and many of the other cases observed since then have revealed a common association with leukopenia and thrombocytopenia [7].

Tn antigen is caused by a hemizygous pleiotropic somatic mutation or gene suppression in adults at the pluripotent stem cell level, creating an abnormal clone in expansion through an autoimmune process, in which it is hypothesized that the patient's Natural Killer cells (NK) target the O-glycans of the normal blood cells and selectively destroy the normal blood cell population. This leads to the loss of anti-Tn and possibly the multi-lineage dysplasia and subsequent cytopenias [8].

Further studies have shown that TnP results from the inactivation of C1GALT1C1, a gene located at Xq24, which encodes a chaperone required for the correct functioning of T-synthase (syn.Gal-b-1-3transferase), a key enzyme in the synthesis of O-glycans. This results in the exposure of GalNAc-linked to serine or threonine on polypeptide backbones, exposing the otherwise cryptic Tn antigen to the erythrocyte surface [8].

A diagnosis of TnP is made using mixed-field seed lectin agglutination. Lectin typing reagents contain proteins that recognize specific carbohydrates on RBC membranes, causing their direct agglutination. Positive agglutination reactions following exposure of the patient's red blood cells to specific lectins derived from seeds of *Dolichos biflorus, Glycine soja *and *Salvia sclerea *plant species are considered as pathognomonic for diagnosis (Table 1). The only exception occurs in patients with blood group A, due to its chemical similarity with the Tn antigen [1-5,9]; monoclonal anti-Tn reagents are available for these cases [9]. Many other types of agglutination have been described in the literature. Most of them are transient and related to episodes of acute infection where bacterial enzymes (for example, neuraminidase and endoβ-galactosidase) expose such antigens (T, Th, Tk, TX, VA). There are some other inherited types of agglutination whose frequency has not been established (Cad [Sda], HEMPAS, NOR, Hyde Park, Tr) [9-11].

**Table 1:** Mixed field polyagglutination reactions with specific seed lectins

*Mixed field polyagglutination reactions with specific seed lectins*									
	**T**	**Tn**	**Th**	**Tk**	**Tx**	**Hyde Park**	**VA**	**Cad**	**NOR**

***Leonurus cardiaca***	-	-	-	-	-	-	-	+	-

***Dolichos biflorus***	-	+	-	-	-	-	-	+	-

***Glycine soja***	+	+	-	-	-	+/-	-	+	-

***Vicia cretia***	+	-	+	-	-	Weak	-	-	-

***Griffonia simplicifolla II***	-	-	-	+	-	+	-	-	-

***Arachis hypogaea***	+	-	+	+	+	Weak	-	-	-

***Salvia sclerea***	-	+	-	-	-	-	-	-	-

***Salvia horminum***	-	+	-	-	-	-	-	-	-

TnP is not directly linked to malignancy or an increased risk of invasive infections, with many reports of elderly Tn individuals in apparently good health. However, the Tn antigen itself has been widely studied as a marker of tumor cells: O-glycans are common constituents of membranes and secreted mucins and an exposure of Tn and sialyl-Tn has been demonstrated in many carcinomas [3].

## Conclusions

To the best of our knowledge, this is the first report of TnP in the context of febrile neutropenia. The recognition of TnP has important clinical implications, because of its benign natural history. An awareness of this fact decreases the need for invasive and costly medical interventions, even though close monitoring is advised in the event of surgery to avoid major complications. As a teaching point, we should include this diagnosis in our differential whenever we encounter a diagnosis of refractory long-standing idiopathic cytopenia and inconclusive bone marrow results displaying multi-lineage dysplasia. The management of this condition is conservative. Blood count levels should be obtained every 6 to 12 months, and a bone marrow biopsy is warranted if there is a significant change in the baseline numbers, frequent fevers, infections or hemorrhagic events.

No additional measures are required for patients with TnP during a blood product transfusion. The passive transfer of anti-Tn is unlikely to be hazardous because donor anti-Tn would be diluted with anti-coagulant, and IgM is unlikely to cause *in vivo *hemolysis. It is also clear that TnP patients should not be candidates for blood donation [1,12].

The infrequency with which TnP is encountered, its clinical features and its pathophysiology makes it a formidable diagnostic challenge.

## Consent

Written informed consent was obtained from the patient for publication of this case report and any accompanying images. A copy of the written consent is available for review by the Editor-in-Chief of this journal.

## Competing interests

The authors declare that they have no competing interests.

## Authors' contributions

ALB and DH treated the patient, analyzed and interpreted the patient data and wrote the manuscript. SS, AB, GT and CK were major contributors to the literature research and in writing the manuscript. All authors read and approved the final manuscript.
